# Subregional neuromelanin-sensitive MRI of the substantia nigra is associated with striatal dopaminergic function in early-stage Parkinson's disease

**DOI:** 10.3389/fnagi.2026.1980030

**Published:** 2026-10-28

**Authors:** Yun Hwa Roh, Wonseok Whi, Seung Hwan Moon, Hwan Heo, Soohwa Song, Beomseok Sohn

**Affiliations:** 1 Department of Radiology, Samsung Medical Center, Sungkyunkwan University School of Medicine, Seoul, Republic of Korea; 2 Department of Radiology and Research Institute of Radiology, Asan Medical Center, University of Ulsan College of Medicine, Seoul, Republic of Korea; 3 Department of Nuclear Medicine, Samsung Medical Center, Sungkyunkwan University School of Medicine, Seoul, Republic of Korea; 4 Heuron Co., Ltd., Seoul, Republic of Korea

**Keywords:** ^18^F-FP-CIT PET, dopamine transporter imaging, neuromelanin-MRI, Parkinson’s disease, positron emission tomography, substantia nigra pars compacta

## Abstract

Neuromelanin-sensitive magnetic resonance imaging (NM-MRI) enables radiation-free assessment of nigral dopaminergic integrity in Parkinson's disease (PD), but its quantitative relationship with striatal dopaminergic function has not been well established. We therefore investigated whether NM-MRI-derived volumes of the substantia nigra pars compacta (SNpc) are associated with striatal dopamine transporter availability measured by ^18^F-FP-CIT positron emission tomography (PET) in early-stage PD. In this retrospective study, consecutive patients with early-stage PD who underwent both NM-MRI and ^18^F-FP-CIT PET between October 2021 and May 2023 were identified, and those with an inter-scan interval exceeding 90 days were excluded. NM-MRI-derived volumes of the associative, limbic, and sensorimotor SNpc subregions were quantified, and striatal dopaminergic function was assessed as specific binding ratios (SBRs) of the ventral and dorsal striatum, striatal mean standardized uptake value, striatal volume, and total dopamine transporter activity. Univariate and multivariable linear regression models incorporating subregion volume, age, and sex were used to relate SNpc subregion volumes to PET measures. Among 136 patients (mean age, 67.5 ± 9.0 years; 81 men [59.6%]), the limbic subregion showed the strongest associations with all ^18^F-FP-CIT PET measures, with the greatest explanatory power for ventral striatal SBR (*R*^2^ = 0.276, *p* < 0.001) *and dorsal striatal SBR* (*R*^2^ = 0.225, *p* < 0.001). Multivariable models incorporating limbic subregion volume, age, and sex yielded adjusted *R*^2^ values of 0.472 and 0.318 for ventral and dorsal striatal SBRs, respectively, with limbic subregion volume remaining independently associated with both models (*p* < 0.001). In repeated 5-fold cross-validation, these models yielded cross-validated *R*^2^ values of 0.449 and 0.287, respectively, and were higher than those of corresponding models based on whole-SNpc volume. These findings suggest that limbic SNpc subregion volume on NM-MRI is associated with striatal dopaminergic function in early-stage PD, supporting NM-MRI as a radiation-free complement to dopamine transporter PET.

## 1 Introduction

Parkinson’s disease (PD) is a progressive neurological disorder primarily characterized by motor symptoms, including bradykinesia, rigidity, and resting tremor, resulting from degeneration of dopaminergic neurons in the substantia nigra (SN) (Braak et al., 2003; Hodaie et al., 2007; Morris et al., 2024). Dopaminergic imaging plays a central role in the diagnostic evaluation of PD. In particular, ^18^F-FP-CIT positron emission tomography (PET) enables visualization of presynaptic dopamine transporter availability with high sensitivity and specificity and is widely used to confirm nigrostriatal degeneration in clinically uncertain cases (Kagi et al., 2010; Sung et al., 2024). However, ^18^F-FP-CIT PET has several limitations, including radiation exposure, high cost, and the need for specialized nuclear medicine infrastructure and expertise for radiopharmaceutical production. Given these limitations, the development of a noninvasive, widely accessible magnetic resonance imaging (MRI)-based alternative for estimating dopaminergic function would have considerable clinical utility, particularly for the detection of early-stage disease.

Neuromelanin-sensitive MRI (NM-MRI) offers such a noninvasive approach for assessing dopaminergic neuron loss in PD. Neuromelanin (NM) is a pigment found in dopaminergic neurons of the SN pars compacta (SNpc) that generates MRI contrast through its paramagnetic binding to iron. NM signal diminishes as these neurons degenerate, providing a structural correlate of disease progression (Sulzer et al., 2018). NM-MRI enables parcellation of the SNpc into three functional subregions, namely, associative, limbic, and sensorimotor, each connected to specific striatal regions that receive input from corresponding frontal cortical areas (Haber, 2003; Zhang et al., 2017; Biondetti et al., 2021).

While previous studies have investigated the relationship between NM-MRI signal and dopamine transporter imaging using single-photon emission computed tomography, these analyses were largely based on whole-SNpc measurements. Because the SNpc subregions may degenerate at different rates (Biondetti et al., 2020; Biondetti et al., 2021), whole-region measurement may dilute the relationship between nigral structure and striatal dopaminergic function. Subregional analysis is therefore needed to determine how closely NM-MRI reflects striatal dopaminergic function. PET-based analyses, particularly those employing ^18^F-FP-CIT, also remain limited, and to our knowledge, no previous study has systematically examined the quantitative association between subregional NM-MRI-derived volumes of SNpc subregions and PET-derived dopaminergic activity in early-stage PD. Establishing such structure-function correlations may facilitate the development of MRI-based imaging biomarkers that could complement functional PET imaging, particularly in settings where access to nuclear medicine is limited.

This study aimed to investigate whether subregional NM-MRI-derived SNpc volumes were associated with striatal dopaminergic function, as assessed by ^18^F-FP-CIT PET, in patients with early-stage PD.

## 2 Materials and methods

### 2.1 Study population

This study was conducted in accordance with the ethical principles of the Declaration of Helsinki. This retrospective study was approved by the Institutional Review Board of Samsung Medical Center (IRB No. 2025-09-181), and the requirement for informed consent was waived because of the retrospective study design.

Consecutive patients with PD who underwent both NM-MRI and ^18^F-FP-CIT PET between October 2021 and May 2023 were retrospectively identified by searching the institutional database of a single tertiary medical center. All participants met the following criteria: (1) age > 18 years; (2) diagnosis of PD based on the clinical diagnostic criteria of the Movement Disorders Society (Postuma et al., 2015), established by an experienced movement-disorder neurologist; (3) early-stage disease, defined as a Hoehn and Yahr scale score < 3 and disease duration ≤60 months; (4) absence of concomitant neurological or psychiatric disorders; and (5) no history of intracranial surgery or traumatic brain injury. Patients were excluded if the interval between MRI and ^18^F-FP-CIT PET exceeded 90 days, or if errors were identified during quality control of the automated segmentation step

Disease severity was evaluated using the Movement Disorder Society-Unified Parkinson's Disease Rating Scale (MDS-UPDRS) Part III score (Goetz et al., 2012) and the Hoehn and Yahr scale (Hoehn and Yahr, 1967).

Two independent supplementary cohorts from the same institution were included for contextual comparison: an NM-MRI cohort (*n* = 357) for subregional volume comparison and an ^18^F-FP-CIT PET cohort (*n* = 197) for comparison of the regional distribution of striatal dopamine transporter binding. Each cohort comprised patients with early-stage PD and disease controls, defined as patients evaluated for suspected parkinsonism but ultimately diagnosed with non-parkinsonian conditions by board-certified neurologists. Detailed cohort characteristics are provided in the Supplementary tables.

### 2.2 MRI acquisition

All MRI scans were performed using a 3-T scanner (Ingenia 3.0 T CX; Philips Healthcare, Best, the Netherlands). The imaging protocol included the following sequences:

A three-dimensional (3D) T1-weighted turbo field echo sequence was acquired with the following parameters: field of view = 240 × 240 mm^2^, flip angle = 8°, voxel size = 1 × 1 × 1 mm^3^, echo time/repetition time = 4.6/9.9 ms, and scanning time = 3 min 29 s.

3D multi-echo gradient echo-based NM-sensitive images were acquired using the SandwichNM protocol (Ji et al., 2022) with the following parameters: field of view = 256 × 180 mm^2^, flip angle = 20°, voxel size = 0.8 × 0.8 × 1.2 mm^3^, echo times = 4.1, 11.3, 18.6, and 25.8 ms, repetition time = 60 ms, and scanning time = 5 min 10 s.

### 2.3 NM-MRI analysis

Image preprocessing and registration followed the pipeline reported in a previous study (Roh et al., 2026). In brief, NM contrast within the SNpc was enhanced by voxel-wise multiplication of the two echoes acquired at 4.1 and 11.3 ms, and noise was suppressed with a Tukey window. Skull stripping (BEaST) (Eskildsen et al., 2012), tissue probability map generation (CAT) (Farokhian et al., 2017), and N4 bias field correction (Tustison et al., 2010) of the T1-weighted (T1w) and NM images were then performed. Labels for the midbrain, pons, thalamus, third ventricle, and ventral diencephalon were obtained from the T1w images using a Swin UNETR segmentation network (Hatamizadeh et al., 2022) trained on 190 manually annotated T1w images (155 training, 35 validation). Registration relied on the SyN algorithm in ANTs (Tustison et al., 2021) (version 2.6.0): individual brain masks were affinely aligned to an NM template generated using the method previously described (Sung et al., 2021) and the NM images transformed into template space, voxels unmatched between individual images and the template were excluded before nonlinear registration, and template-space masks of the periaqueductal gray (PAG), SNpc, and its associative, limbic, and sensorimotor subregions were propagated into individual space.

NM volume was quantified using a threshold anchored to the PAG reference region to account for intensity variation across acquisitions (Cassidy et al., 2019). The equation used to calculate the threshold value for quantifying NM volume in the SNpc is defined as:


$$\text{Threshold}=2.94\times m(V_{\mathrm{PAG}})+0.23^{*}\;\sigma (V_{\mathrm{PAG}})$$

Where $V_{\mathrm{PAG}}$ is the voxel set within the PAG mask, and m() and σ() are the mean and standard deviation of the intensity values, respectively; and 2.94 and 0.23 are the optimal weights established experimentally. The NM volume was calculated using the following equation:


$$NM_{\mathrm{vol}}=|\{\;v\in V_{\mathrm{FG}}v\geq \text{Threshold}\}|\times s(V_{\mathrm{FG}})$$

Where $V_{\mathrm{FG}}$ is the target foreground region (total SNpc or its associative, limbic, or sensorimotor subregion) and $s(V_{\mathrm{FG}})$ is the cumulative voxel-spacing product. NM volumes were normalized to intracranial volume, estimated from the T1w images by a previously described method (Khalili et al., 2019), and multiplied by 10^4^.

Quality control was performed after image processing. Placement of the SNpc subregion and PAG masks in individual space was visually inspected in all patients, with reference to the midbrain anatomy on the co-registered T1-weighted images. Scans with misplaced masks were classified as processing failures and excluded.

### 2.4 ^18^F-FP-CIT PET acquisition

All study participants underwent ^18^F-FP-CIT PET/computed tomography (CT) scans using the Discovery STe scanner (GE Healthcare, Milwaukee, WI, USA). No intravenous or oral contrast agents were administered. Patients were instructed to withhold medications that could interfere with ^18^F-FP-CIT uptake. Imaging was performed 120 min after intravenous injection of ^18^F-FP-CIT (185 MBq ± 5%).

Brain CT was performed using a 16-slice helical CT scanner (120 kVp, 50 mAs) for attenuation correction, followed by a 10-min PET scan in 3D mode. PET images (voxel size: 3.9 × 3.9 × 3.3 mm^3^) were reconstructed using the ordered-subsets expectation maximization algorithm (20 subsets, 2 iterations).

### 2.5 Quantitative analysis of dopamine transporter imaging

The volume, striatal mean standardized uptake value (SUVmean), and total dopamine transporter activity (TDA), defined as the product of volume and SUVmean, of the bilateral striatum were measured using Volume Viewer software (GE Advantage Workstation 4.7), which automatically delineates a volume of interest (VOI) using an isocontour threshold method based on the SUV. The occipital cortex was used as the reference region for determining the VOI boundary (Jeong et al., 2013). TDA was calculated by multiplying SUVmean by the volume as a quantitative, volume-dependent (or extensive) metric.

Specific binding ratios (SBRs) of ^18^F-FP-CIT in striatal and extrastriatal subregions, including the dorsal striatum, ventral striatum, caudate nucleus, putamen (all subdivided into anatomically defined subregions), and pallidum, as well as the dorsal raphe nucleus, locus coeruleus, substantia nigra, and subthalamic nucleus, were quantified using deep learning-based software (BTXBrain DAT PET, Korea), which enables MRI-free spatial normalization (Kang et al., 2024). SBRs were obtained from VOIs defined on atlas templates from the Melbourne Striatal for CIT, with the occipital region serving as the reference region. The overall workflow for image processing and analysis is provided in Figure 1.

**Figure 1:**
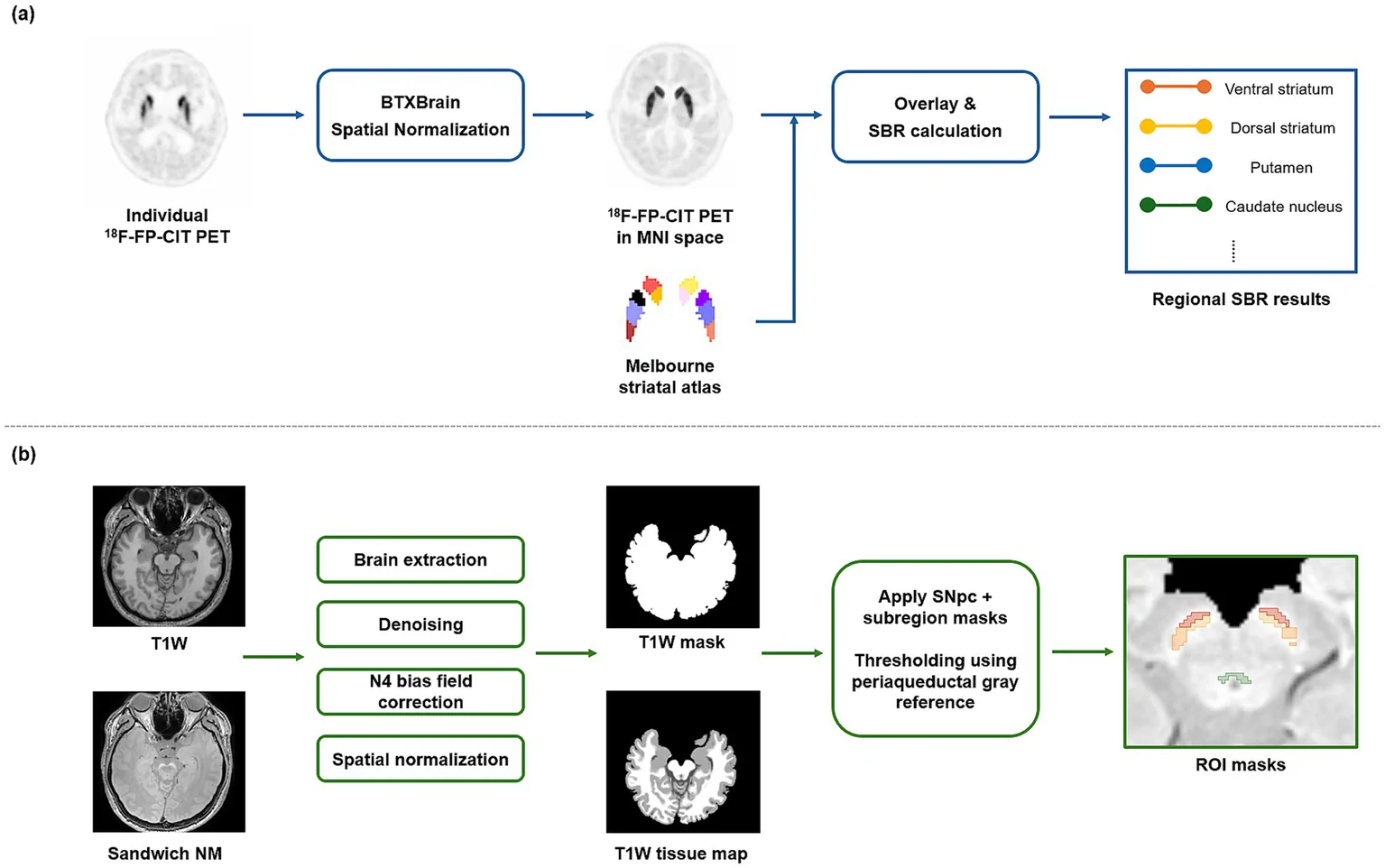
Schematic workflow of **(a)** ^18^F-FP-CIT PET and **(b)** NM-MRI image analysis. NM-MRI, neuromelanin-sensitive magnetic resonance imaging; SBR, specific binding ratio; T1W, T1-weighted imaging; ROI, region of interest.

### 2.6 Statistical analysis

Descriptive statistics were presented as mean ± standard deviation for continuous variables and frequency (percentage) for categorical variables. Normality of the data distribution was assessed using the Shapiro-Wilk test.

For the primary analysis, univariate linear regression analyses were first performed using each NM-MRI-derived SNpc subregion volume (associative, limbic, sensorimotor) as a predictor of striatal PET variables (bilateral striatal volume, SUVmean, and TDA, as well as dorsal and ventral striatal SBRs). To account for potential demographic confounding, additional univariate analyses were performed to assess the associations of age and sex with ventral and dorsal striatal SBRs, which showed the strongest associations with the SNpc subregions in the previous analysis. The effects of age and sex on SNpc subregion volumes were also examined to characterize demographic influences on these measurements. Based on the univariate analysis results, multivariable linear regression models were constructed using limbic subregion volume, age, and sex as independent variables to estimate ventral and dorsal striatal SBRs. The added value of limbic subregion beyond demographic variables was assessed by comparing a model including age and sex alone with a model additionally including limbic subregion volume, using the change in adjusted *R*^2^ and a partial *F*-test. Regression coefficients (*β*) are presented as unstandardized coefficients (per unit of normalized subregion volume, per 1-year increase for age, and female relative to male for sex).

To evaluate the added value of subregional analysis, whole-SNpc volume and limbic subregion volume were compared using the same modeling strategy: univariate models and multivariable models adjusted for age and sex, fitted in the same sample for ventral and dorsal striatal SBRs. Model performance was compared using *R*^2^ and adjusted *R*^2^ (*R*^2^ adjusted for the number of predictors).

Internal validation was performed using 5-fold cross-validation repeated 100 times, and cross-validated *R*^2^, mean absolute error (MAE), and root mean squared error (RMSE) were reported. Because the limbic subregion and the ventral and dorsal striatal SBR outcomes were chosen from the univariate analyses, cross-validation evaluated the performance of the final regression models, not the whole selection process.

For the secondary analysis, univariate linear regression analyses were additionally performed between each SNpc subregion volume and the SBRs of detailed striatal and extrastriatal regions. *p*-values for the associations between SNpc subregion volumes and PET measures were corrected for multiple comparisons using the Benjamini-Hochberg false discovery rate, applied separately to the primary striatal analyses and to the detailed regional analyses.

For the supplementary comparisons, between-group differences in NM-MRI-derived SNpc subregion volumes and ^18^F-FP-CIT SBRs between patients with early-stage PD and disease controls were assessed using age- and sex-adjusted analysis of covariance.

All statistical analyses were performed using R (version 4.2.1; R Foundation for Statistical Computing, Vienna, Austria). All tests were two-sided, and a *p*-value < 0.05 was considered statistically significant.

## 3 Results

### 3.1 Baseline characteristics

Among the 194 patients with early-stage PD who underwent both NM-MRI and ^18^F-FP-CIT PET, 58 were excluded because the interval between the two imaging studies exceeded 90 days. A total of 136 patients were included in the final analysis. The demographic and clinical characteristics of the study participants are summarized in Table 1.

**Table 1:** Baseline characteristics of the study participants (*n* = 136).

Characteristic	Value
Age, years	67.5 ± 9.0
Male sex, n (%)	81 (59.6)
Disease duration, months	20.1 ± 15.1
Hoehn and Yahr scale, score	1.6 ± 0.5
MDS-UPDRS III	17.3 ± 9.2
MRI to UPDRS interval, months	2.0 ± 2.2
MRI to PET interval, days	−6.2 ± 32.2
SNpc subregion volumes, normalized to ICV (×10^4^)	
Limbic	0.48 ± 0.14
Associative	0.32 ± 0.14
Sensorimotor	0.44 ± 0.12
^18^F-FP-CIT PET striatal measures	
Striatal SUVmean	3.96 ± 0.65
Striatal volume, cm^3^	39.83 ± 8.27
Striatal TDA	159.96 ± 49.39
SBR of dorsal striatum	2.07 ± 0.68
SBR of ventral striatum	3.18 ± 0.97

Data are presented as mean ± standard deviation or *n* (percentage).

MDS-UPDRS, Movement Disorder Society-Unified Parkinson's Disease Rating Scale; SNpc, substantia nigra pars compacta; PET, positron emission tomography; SUV, standardized uptake value; TDA, total dopamine activity; SBR, specific binding ratio.

The mean age was 67.5 ± 9.0 years, and 81 patients (59.6%) were male. The mean disease duration was 20.1 ± 15.1 months, and the mean Hoehn and Yahr scale score was 1.6 ± 0.5. The mean MDS-UPDRS Part III score was 17.3 ± 9.2. The mean interval between MRI and clinical motor assessment was 2.0 ± 2.2 months. The mean interval between MRI and PET acquisition was −6.2 ± 32.2 days.

Among the three SNpc subregions identified on NM-MRI, the limbic subregion had the largest mean volume (0.48 ± 0.14), followed by the sensorimotor (0.44 ± 0.12) and associative (0.32 ± 0.14) subregions. The striatal SUVmean was 3.96 ± 0.65, with a mean striatal volume of 39.83 ± 8.27 cm^3^ and a mean TDA of 159.96 ± 49.39. The mean SBR was 3.18 ± 0.97 in the ventral striatum, and 2.07 ± 0.68 in the dorsal striatum.

### 3.2 Association between SNpc subregion volumes on NM-MRI and striatal ^18^F-FP-CIT PET measures

All three SNpc subregions on NM-MRI showed significant associations with multiple ^18^F-FP-CIT PET measures, with varying strengths of association (Table 2). Among the three SNpc subregions, the limbic subregion demonstrated the strongest associations with all ^18^F-FP-CIT PET measures. Limbic subregion volume showed the greatest explanatory power for the ventral striatal SBR (*R*^2^ = 0.276, *p* < 0.001), followed by the dorsal striatal SBR (*R*^2^ = 0.225, *p *< 0.001) and striatal TDA (*R*^2^ = 0.163, *p* < 0.001). The associations with mean striatal volume (*R*^2^ = 0.135, *p *< 0.001) and striatal SUVmean (*R*^2^ = 0.085, *p *< 0.001) were also statistically significant but had lower explanatory power. The sensorimotor and associative region volumes demonstrated weaker associations with striatal ^18^F-FP-CIT PET measures, with *R*^2^ values ranging from 0.036 to 0.109 (all *p *< 0.05). Based on these findings, ventral and dorsal striatal SBRs were selected as the primary outcomes for subsequent multivariable modeling because they showed the strongest associations.

**Table 2:** Univariate regression analysis of associations between SNpc subregion volumes and ^18^F-FP-CIT PET measures.

NM subregions	PET measures	*β*	*p*-value	*R* ^2^
Associative	Striatal SUVmean	1.208	0.003*	0.071
	Striatal volume	12.511	0.014*	0.047
	Striatal TDA	101.439	0.001*	0.086
	SBR of dorsal striatum	1.455	<0.001*	0.095
	SBR of ventral striatum	2.237	<0.001*	0.109
Limbic	Striatal SUVmean	1.388	0.001*	0.085
	Striatal volume	22.366	<0.001*	0.135
	Striatal TDA	146.543	<0.001*	0.163
	SBR of dorsal striatum	2.353	<0.001*	0.225
	SBR of ventral striatum	3.733	<0.001*	0.276
Sensorimotor	Striatal SUVmean	1.034	0.027*	0.036
	Striatal volume	13.788	0.023*	0.04
	Striatal TDA	100.876	0.006*	0.06
	SBR of dorsal striatum	1.405	0.005*	0.062
	SBR of ventral striatum	1.532	0.027*	0.036

*p*-values were corrected for multiple comparisons.

SNpc, substantia nigra pars compacta; PET, positron emission tomography; SUV, standardized uptake value; TDA, total dopamine transporter activity; SBR, specific binding ratio; β, unstandardized regression coefficient; *R*^2^, coefficient of determination.

*indicates statistical significance.

### 3.3 Effects of age and sex on striatal SBR and SNpc subregion volumes

Both age and sex were significantly associated with ventral striatal SBR (Supplementary Table 1). Age was negatively associated with ventral striatal SBR (*β* = −0.059, 95% CI: −0.074 to −0.044, *R*^2^ = 0.299, *p* < 0.001). Female patients had higher ventral striatal SBRs than male patients (*β* = 0.795, 95% CI: 0.488–1.102, *R*^2^ = 0.164, *p* < 0.001). Similar patterns were observed for the dorsal striatum. Age was negatively associated with dorsal striatal SBR (*β* = −0.032, 95% CI: −0.044 to −0.02, *R*^2^ = 0.184, *p* < 0.001), and female patients had higher dorsal striatal SBRs than male patients (*β* = 0.429, 95% CI: 0.207–0.651, *R*^2^ = 0.098, *p *< 0.001).

Age was negatively associated with SNpc subregion volumes (Supplementary Table 2). The strongest association was observed for the limbic subregion (*β* = −0.005, 95% CI: −0.008 to −0.003, *R*^2^ = 0.126, *p* < 0.001), followed by the associative region (*β* = −0.004, 95% CI: −0.007 to −0.001, *R*² = 0.059, *p* = 0.004). The sensorimotor subregion showed a trend toward age-related decline but did not reach statistical significance (*β* = −0.002, 95% CI: −0.004 to 0.0003, *R*² = 0.021, *p* = 00.091). Sex differences were observed in the limbic and associative subregions, with female patients showing significantly larger volumes than male patients (limbic: *β* = 0.076, 95% CI: 0.030–0.121, *R*² = 0.075, *p* = 0.001; associative: *β* = 0.067, 95% CI: 0.019–0.115, *R*² = 0.053, *p* = 0.007). No significant sex difference was observed in the sensorimotor region (*p* = 0.355).

### 3.4 Multivariable models for ventral and dorsal striatal SBRs

Based on the univariate analysis identifying the limbic subregion showing the strongest association and confirming the effects of age and sex, multivariable models incorporating limbic subregion volume, age, and sex were constructed (Table 3). The multivariable model had an adjusted *R*^2^ of 0.472 for ventral striatal SBR. Limbic subregion volume (*β* = 2.23, 95% CI: 1.26–3.2, *p* < 0.001), age (*β* = −0.043, 95% CI: −0.057 to −0.028, *p* < 0.001), and sex (*β* = 0.506, 95% CI: 0.253–0.759, *p* < 0.001) were each independently associated with ventral striatal SBR.

**Table 3:** Multivariable regression models for striatal SBRs.

Outcome	Variables	*β* (95% CI)	*P*-value	Adjusted *R*^2^	CV *R*^2^	CV MAE	CV RMSE
SBR of ventral striatum	Limbic subregion	2.23 (1.26, 3.20)	<0.001*	0.472	0.449	0.558	0.716
	Age	−0.043 (−0.057, −0.028)	<0.001*				
	Sex (female)	0.506 (0.253, 0.759)	<0.001*				
SBR of dorsal striatum	Limbic subregion	1.60 (0.831, 2.369)	<0.001*	0.318	0.287	0.436	0.568
	Age	−0.022 (−0.033, −0.010)	0.001*				
	Sex (female)	0.250 (0.046, 0.448)	0.016*				

SBR, specific binding ratio; *β*, unstandardized regression coefficient; CI, confidence interval; *R*^2^, coefficient of determination; CV, cross-validated (5-fold, repeated 100 times); MAE, mean absolute error; RMSE, root mean squared error.

*indicates statistical significance.

The multivariable model for the dorsal striatum had an adjusted *R*^2^ of 0.318, with independent associations for limbic subregion volume (*β* = 1.6, 95% CI: 0.831–2.369, *p *< 0.001), age (*β* = −0.022, 95% CI: −0.033 to −0.01, *p *= 0.001), and sex (*β* = 0.250, 95% CI: 0.046–0.448, *p* = 0.016).

The performance of the multivariable models are illustrated in Figure 2. Compared with the univariate model including limbic subregion volume alone (ventral striatum: adjusted *R*^2^ = 0.269; dorsal striatum: adjusted *R*^2^ = 0.219), the multivariable models showed substantial improvements in model fit, with increases in adjusted *R*^2^ of 0.203 and 0.099 for the ventral and dorsal striatum, respectively. In repeated 5-fold cross-validation, the multivariable models yielded cross-validated *R*^2^ values of 0.449 and 0.287, MAE of 0.558 and 0.436, and RMSE of 0.716 and 0.568 for ventral and dorsal striatal SBRs, respectively (Table 3).

**Figure 2:**
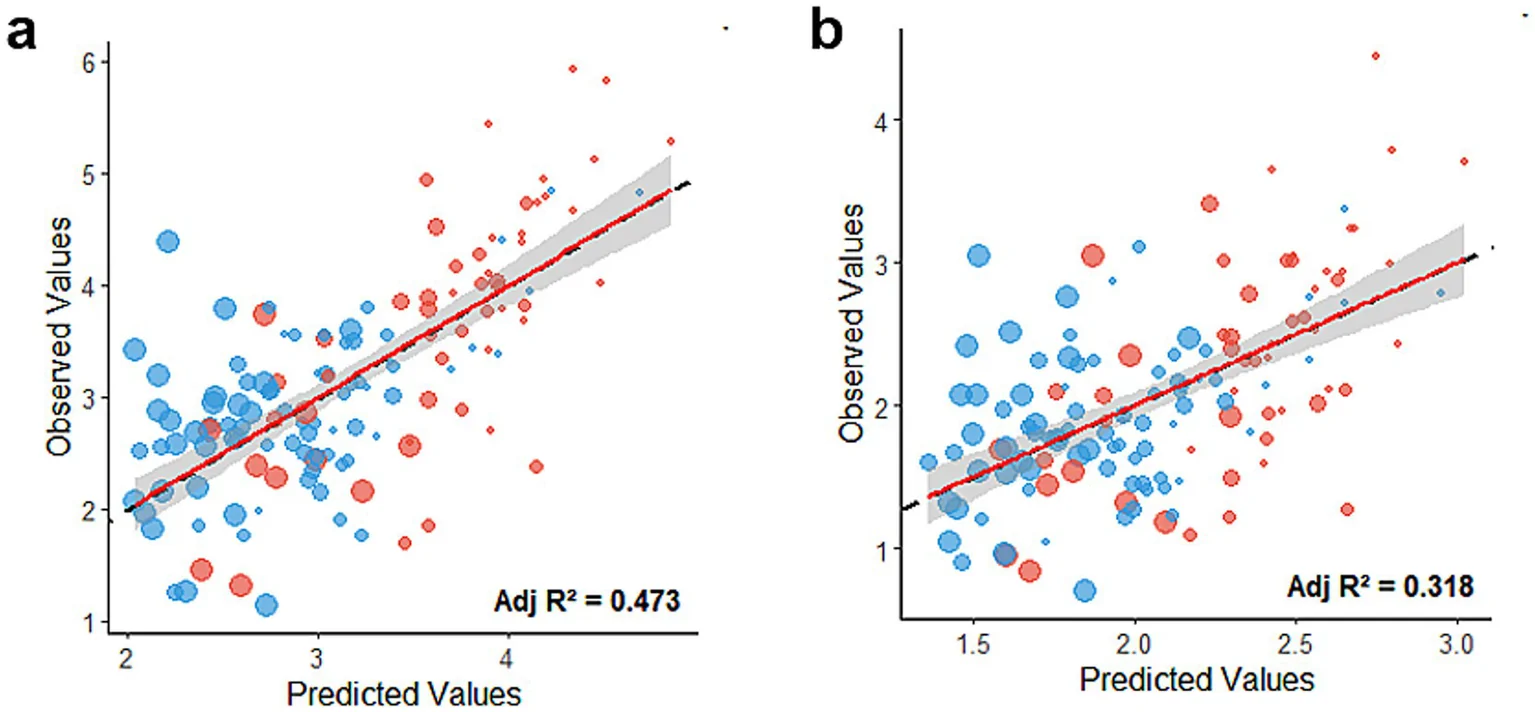
Performance of multivariable models for striatal SBR predicted versus observed values from multivariable linear regression models incorporating limbic subregion volume, age, and sex for **(a)** ventral and **(b)** dorsal striatal SBRs. Points are colored according to sex (blue = male; red = female) and sized according to age quartiles (larger points indicate older age). The dashed diagonal line represents perfect agreement (*y* = *x*), whereas the red line represents the fitted relationship between the predicted and observed values. SBR, specific binding ratio.

Compared with a model including age and sex alone, adding limbic subregion volume increased adjusted *R*^2^ from 0.395 to 0.472 for ventral striatal SBR (*p* < 0.001) and from 0.236 to 0.318 for dorsal striatal SBR (*p* < 0.001), with corresponding increases in cross-validated *R*^2^ (ventral, 0.371 to 0.449; dorsal, 0.203 to 0.287) (Supplementary Table 3).

Representative cases illustrating the association between limbic subregion volume and striatal dopamine transporter binding are shown in Figure 3.

**Figure 3:**
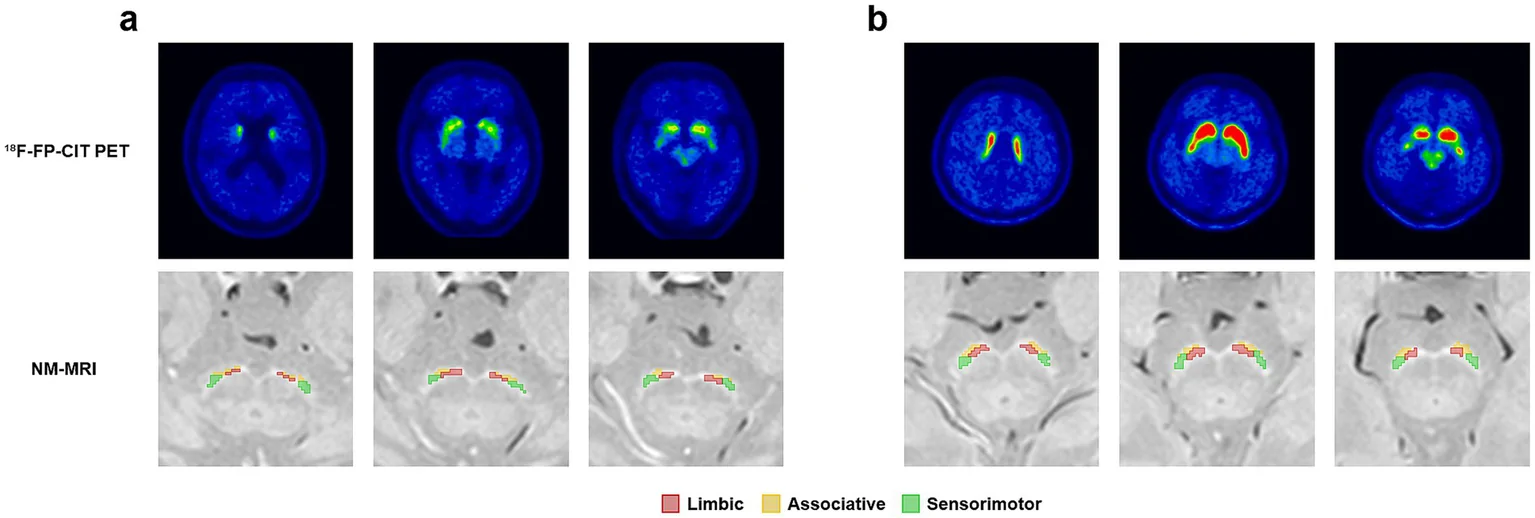
Representative cases illustrating the association between SNpc subregion volumes on NM-MRI and striatal dopamine transporter binding on ^18^F-FP-CIT PET. **(a)** A patient with a low limbic region volume (0.18, normalized to ICV), a dorsal striatal SBR of 1.21, and a ventral striatal SBR of 1.85. **(b)** A patient with a high limbic subregion volume (0.70, normalized to ICV), a dorsal striatal SBR of 3.70, and a ventral striatal SBR of 5.27. SNpc, substantia nigra pars compacta; NM-MRI, neuromelanin-sensitive MRI; SBR, specific binding ratio.

### 3.5 Comparison of whole-SNpc and limbic subregion volumes

Compared with whole-SNpc volume, limbic subregion volume showed higher explanatory power for both ventral (*R*^2^ = 0.276 vs. 0.155) and dorsal striatal SBRs (*R*^2^ = 0.225 vs. 0.149) (Supplementary Table 4). This pattern persisted in the age- and sex-adjusted models (adjusted *R*^2^, ventral: 0.472 vs. 0.428; dorsal: 0.318 vs. 0.286) and in repeated 5-fold cross-validation (cross-validated *R*^2^, ventral: 0.449 vs. 0.400; dorsal: 0.287 vs. 0.250).

### 3.6 Detailed regional analysis of SNpc subregions and PET subregions

To further characterize the regional specificity of NM-MRI–PET associations beyond the primary striatal targets, the relationships between SNpc subregion volumes and SBRs across additional striatal and extrastriatal subregions were evaluated (Supplementary Table 5). Among the striatal PET subregions, the strongest associations were observed in the caudate nucleus. Limbic subregion volume showed the strongest association with the dorsal anterior caudate nucleus (*R*^2^ = 0.284, *p* < 0.001). The putamen subregions showed lower effect sizes, with the limbic subregion demonstrating the strongest association with the ventral anterior putamen (*R*^2^ = 0.213, *p *< 0.001).

The associations between SNpc subregion volumes and PET extrastriatal regions were weaker than those observed in the striatal regions, with *R*^2^ values ranging from <0.001 to 0.1. Most PET brainstem regions showed no significant associations with subregion volumes, except for the substantia nigra, which showed a weak association with limbic subregion volume (*R*^2^ = 0.039, *p *= 0.036).

## 4 Discussion

Subregional volumes of the SNpc using NM-MRI, particularly those of the limbic subregion, were associated with striatal dopaminergic function in patients with early-stage PD. Among the three SNpc subregions examined, the limbic subregion showed the strongest associations with ^18^F-FP-CIT PET measures, with *R*^2^ values of 0.276 for ventral striatal SBR and 0.225 for dorsal striatal SBR. When combined with the demographic variables age and sex in multivariable models, the adjusted *R*^2^ increased to 0.472 for ventral striatal SBR and 0.318 for dorsal striatal SBR.

Prior studies have established that NM-MRI of the SNpc reflects presynaptic dopaminergic loss, although findings vary according to imaging modality. Single-photon emission computed tomography studies have linked NM-positive SNpc volume and MRI-based deep learning models to ^123^I-FP-CIT SBRs (Kuya et al., 2016; Bae et al., 2023), whereas PET studies using ^11^C-PE2I and ^18^F-FP-CIT have reported associations between NM-MRI measures and striatal DAT uptake in early-stage PD (Martin-Bastida et al., 2019; Park et al., 2023). However, most previous analyses relied on whole-SNpc regions of interest, limiting anatomical specificity and precluding assessment of the differential contributions of functionally distinct nigral subregions (Sulzer et al., 2018). By quantifying volumetric measures at the SNpc subregion level, the present study extends these findings by providing a more anatomically specific characterization of subregional nigrostriatal structure-function relationships. Moreover, limbic subregion volume showed higher adjusted *R*^2^ and cross-validated *R*^2^ than whole-SNpc volume, although this difference was not formally tested.

The association between limbic subregion volume and ventral striatal SBR (*R*^2^ = 0.276) aligns well with established nigrostriatal connectivity patterns, in which projections from the medial SNpc to the ventral striatum are anatomically prominent (Haber et al., 2000; McGregor and Nelson, 2019). In contrast, the weaker association between the sensorimotor SNpc subregion and dorsal striatal SBR may be attributable to several factors. From a temporal staging perspective, presynaptic DAT loss detectable by PET may precede somatic neuronal degeneration visible on NM-MRI, consistent with the “dying-back” pattern of axonal pathology, in which terminal dysfunction occurs before nigral cell body loss (Caminiti et al., 2017; Fazio et al., 2018). A contributing technical factor may be the higher iron concentrations and lower signal-to-noise ratio in the ventrolateral midbrain, which may reduce NM-MRI sensitivity, particularly in the sensorimotor subregion (Martin et al., 2008; Biondetti et al., 2020).

These findings should not be interpreted as indicating that the limbic compartment is uniquely or selectively vulnerable in early-stage PD. Rather, they suggest that, by the time of clinical presentation, degeneration in the limbic subregion has progressed sufficiently to parallel dopaminergic loss measurable on PET, resulting in a stronger structure-function association than that observed for the other subregions. A parallel vulnerability framework, in which all three SNpc subregions are affected early but differ in the extent to which structural loss corresponds to functional PET abnormalities, provides a more coherent interpretation of these findings.

To further contextualize these findings, two independent supplementary cohorts comparing patients with early-stage PD and disease controls were examined. In the NM-MRI cohort, the early-stage PD group showed reduced volume across all three SNpc subregions compared with the disease control group (Supplementary Table 6). In a separate ^18^F-FP-CIT PET cohort, striatal dopamine transporter binding was likewise reduced across the measured regions (Supplementary Table 7). Although these MRI and PET cohorts were independent and their measures could not be directly compared, both consistently indicate that structural and functional nigrostriatal involvement is already widespread at the time of clinical presentation. These findings suggest that the differential structure-function associations observed in the primary cohort do not reflect selective involvement of a single subregion but instead arise against a background of diffuse nigrostriatal degeneration, within which the limbic subregion provides the closest structural correlate of striatal dopaminergic function.

From a clinical perspective, these findings suggest that subregional NM-MRI volumes, particularly those of the limbic subregion, could help estimate striatal dopaminergic function in early-stage PD. Although the explained variance of the best-performing model remained moderate (adjusted *R*^2^ = 0.472 and cross-validated *R*^2^ = 0.449 for ventral striatum), indicating that NM-MRI captures only part of the pathophysiology underlying dopaminergic dysfunction, NM-MRI may still serve as a useful adjunct to dopamine transporter imaging because it is radiation-free and widely available, particularly when interpreted alongside clinical assessment in settings where access to nuclear medicine is limited or repeated PET is impractical because of cost or radiation exposure.

Practical implementation also depends on acquisition time. The NM-MRI sequence used in this study required 5 min 10 s, and longer acquisitions are susceptible to motion artifacts in patients with movement disorders. Recent advances in accelerated MRI reconstruction (Qu et al., 2023; Qu et al., 2024; Qu et al., 2026) may help shorten acquisition, and their application to NM-MRI could support wider clinical use.

This study has several limitations. First, the retrospective, single-center design may limit the generalizability of these findings. The NM-MRI acquisition parameters and processing pipeline, although based on validated methods, may yield different quantitative results across scanners, necessitating multicenter validation using standardized protocols. In addition, although mask placement was visually verified in all patients, test-retest imaging was not available, so the scan-rescan reproducibility of the subregion volumes could not be assessed directly. Second, the primary analysis did not include a healthy control group, which would have enabled direct comparison of NM-MRI–PET correlations between patients with early-stage PD and healthy individuals. Third, the cross-sectional design precluded assessment of longitudinal changes in the NM-MRI–PET relationship during disease progression. Future prospective studies incorporating serial imaging at multiple time points are needed to determine whether NM-MRI can reliably track dopaminergic decline and predict clinical progression. Fourth, the limbic subregion and the primary outcomes were chosen from univariate analyses of the full dataset before cross-validation. The cross-validated estimates therefore reflect internal performance of the selected models rather than independent validation of the whole analysis, and external validation is needed.

## 5 Conclusion

Subregional NM-MRI analysis of the SNpc, particularly limbic subregion volume combined with age and sex, showed moderate association with ventral striatal dopaminergic function in early-stage PD. These findings suggest that NM-MRI may complement dopamine transporter PET imaging as a radiation-free and widely accessible measure of nigrostriatal integrity.

## Data Availability

The original contributions presented in the study are included in the article/Supplementary material, further inquiries can be directed to the corresponding author/s.
