# Supplementary material for: Subregional neuromelanin-sensitive MRI of the substantia nigra is associated with striatal dopaminergic function in early-stage Parkinson's disease
Source: Front Aging Neurosci. 2026 Oct 28;18:1980030. doi: 10.3389/fnagi.2026.1980030 (PMC13751972; doi:10.3389/fnagi.2026.1980030)
Supplement: Supplementary file 1 [file Supplementary_file_1.DOCX]

**Supplementary Table 1. Association of age and sex with striatal SBR**

|  | **SBR of Ventral Striatum** | | |  | **SBR of Dorsal Striatum** | | |
| --- | --- | --- | --- | --- | --- | --- | --- |
|  | **β (95% CI)** | **R²** | ***p*-value** |  | **β (95% CI)** | **R²** | ***p*-value** |
| **Age** | -0.059  (-0.074, -0.044) | 0.299 | <0.001* |  | -0.032  (-0.044, -0.02) | 0.184 | <0.001* |
| **Sex (Female)** | 0.795  (0.488, 1.102) | 0.164 | <0.001* |  | 0.429  (0.207, 0.651) | 0.098 | <0.001* |

SBR, specific binding ratio; β, unstandardized regression coefficient; CI, confidence interval; R², coefficient of determination.

* indicates statistical significance.

**Supplementary Table 2. Association of age and sex with SNpc subregion volumes**

|  | **Associative Subregion** | | |  | **Limbic Subregion** | | |  | **Sensorimotor Subregion** | | |
| --- | --- | --- | --- | --- | --- | --- | --- | --- | --- | --- | --- |
|  | **β (95% CI)** | **R²** | ***p*-value** |  | **β (95% CI)** | **R²** | ***p*-value** |  | **β (95% CI)** | **R²** | ***p*-value** |
| **Age** | -0.004  (-0.007, -0.001) | 0.059 | 0.004* |  | -0.005  (-0.008, -0.003) | 0.126 | <0.001* |  | -0.002  (-0.004, 0.0003) | 0.021 | 0.091 |
| **Sex (Female)** | 0.067  (0.019, 0.115) | 0.053 | 0.007* |  | 0.076  (0.030, 0.121) | 0.075 | 0.001* |  | 0.019  (-0.022, 0.061) | 0.006 | 0.355 |

SNpc, substantia nigra pars compacta; β, unstandardized regression coefficient; CI, confidence interval; R², coefficient of determination.

* indicates statistical significance.

**Supplementary Table 3. Comparison of models with and without limbic subregion volume for striatal SBRs**

| **Outcome** | **Model** | **R²** | **Adjusted R²** | **CV**  **R²** | **CV MAE** | **CV RMSE** |
| --- | --- | --- | --- | --- | --- | --- |
| **SBR of ventral striatum** | Age + Sex | 0.404 | 0.395 | 0.371 | 0.595 | 0.765 |
|  | Age + Sex + Limbic subregion | 0.484 | 0.472 | 0.449 | 0.558 | 0.716 |
| **SBR of dorsal striatum** | Age + Sex | 0.247 | 0.236 | 0.203 | 0.461 | 0.600 |
|  | Age + Sex + Limbic subregion | 0.333 | 0.318 | 0.287 | 0.436 | 0.568 |

SBR, specific binding ratio; R², coefficient of determination; CV, cross-validated (5-fold, repeated 100 times); MAE, mean absolute error; RMSE, root mean squared error.

**Supplementary Table 4. Comparison of whole-SNpc and limbic subregion volume models for striatal SBRs**

| **Outcome** | **Model** | **R²** | **Adjusted R²** | **CV R²** | **CV MAE** | **CV RMSE** |
| --- | --- | --- | --- | --- | --- | --- |
| **SBR of ventral striatum** | Whole SNpc | 0.155 | 0.149 | 0.126 | 0.709 | 0.902 |
|  | Limbic subregion | 0.276 | 0.269 | 0.250 | 0.668 | 0.835 |
|  | Whole SNpc + Age + Sex | 0.441 | 0.428 | 0.400 | 0.572 | 0.747 |
|  | Limbic subregion + Age + Sex | 0.484 | 0.472 | 0.449 | 0.558 | 0.716 |
| **SBR of dorsal striatum** | Whole SNpc | 0.149 | 0.143 | 0.119 | 0.493 | 0.631 |
|  | Limbic subregion | 0.225 | 0.219 | 0.199 | 0.476 | 0.602 |
|  | Whole SNpc + Age + Sex | 0.302 | 0.286 | 0.250 | 0.447 | 0.582 |
|  | Limbic subregion + Age + Sex | 0.333 | 0.318 | 0.287 | 0.436 | 0.568 |

SNpc, substantia nigra pars compacta; SBR, specific binding ratio; R², coefficient of determination; CV, cross-validated (5-fold, repeated 100 times); MAE, mean absolute error; RMSE, root mean squared error.

**Supplementary Table 5. Associations between SNpc subregion volumes and ^18^F-FP-CIT PET specific binding ratios**

|  | **SNpc Subregions** | | | | | | | | |
| --- | --- | --- | --- | --- | --- | --- | --- | --- | --- |
|  | **Associative** | | | **Limbic** | | | **Sensorimotor** | | |
| **PET Subregions** | **β** | ***p*-value** | **R²** | **β** | ***p*-value** | **R²** | **β** | ***p*-value** | **R²** |
| **Striatal Subregions** |  |  |  |  |  |  |  |  |  |
| Caudate nucleus | 2.331 | **<0.001*** | 0.119 | 3.78 | **<0.001*** | 0.283 | 1.593 | **0.036*** | 0.039 |
| Caudate DA | 2.549 | **<0.001*** | 0.119 | 4.138 | **<0.001*** | 0.284 | 1.802 | **0.031*** | 0.042 |
| Caudate VA | 2.409 | **<0.001*** | 0.118 | 3.863 | **<0.001*** | 0.273 | 1.449 | **0.067** | 0.03 |
| Caudate tail | 1.736 | **<0.001*** | 0.107 | 2.749 | **<0.001*** | 0.243 | 1.369 | **0.022*** | 0.047 |
| Caudate body | 2.142 | **<0.001*** | 0.113 | 3.54 | **<0.001*** | 0.277 | 1.52 | **0.035*** | 0.04 |
| Putamen | 1.03 | **0.009*** | 0.06 | 1.662 | **<0.001*** | 0.141 | 1.314 | **0.006*** | 0.068 |
| Putamen DA | 1.301 | **0.006*** | 0.069 | 2.046 | **<0.001*** | 0.155 | 1.514 | **0.007*** | 0.066 |
| Putamen VA | 1.945 | **<0.001*** | 0.094 | 3.089 | **<0.001*** | 0.213 | 1.642 | **0.022*** | 0.047 |
| Putamen DP | 0.589 | 0.092 | 0.026 | 0.974 | **0.007*** | 0.063 | 1.111 | **0.007*** | 0.064 |
| Putamen VP | 0.465 | 0.161 | 0.017 | 0.811 | **0.022*** | 0.048 | 1.069 | **0.007*** | 0.064 |
| **Extrastriatal Regions** |  |  |  |  |  |  |  |  |  |
| Pallidum | 0.138 | 0.116 | 0.022 | 0.308 | **<0.001*** | 0.099 | 0.191 | 0.067 | 0.03 |
| Anterior pallidum | 0.159 | 0.125 | 0.021 | 0.368 | **<0.001*** | 0.1 | 0.204 | 0.101 | 0.024 |
| Posterior pallidum | 0.113 | 0.125 | 0.021 | 0.236 | **<0.001*** | 0.081 | 0.175 | **0.03*** | 0.035 |
| Dorsal raphe nucleus | 0.001 | 0.992 | <0.001 | 0.121 | 0.414 | 0.005 | 0.037 | 0.825 | <0.001 |
| Locus coeruleus | -0.015 | 0.872 | <0.001 | 0.121 | 0.222 | 0.011 | -0.089 | 0.431 | 0.005 |
| Substantia nigra | 0.083 | 0.308 | 0.008 | 0.195 | **0.022*** | 0.039 | 0.145 | 0.134 | 0.017 |
| Subthalamic nucleus | 0.026 | 0.741 | 0.001 | 0.071 | 0.384 | 0.006 | 0.081 | 0.38 | 0.006 |

*p*-values were corrected for multiple comparisons.

SNpc, substantia nigra pars compacta; β, unstandardized regression coefficient; R², coefficient of determination; DA, dorsal anterior; VA, ventral anterior; DP, dorsal posterior; VP, ventral posterior.

* indicates statistical significance.

**Supplementary Table 6.** **Comparison of SNpc subregional volumes between disease controls and patients with early-stage PD**

|  | **Disease Controls** | **Early PD** | **Reduction (%)** | ***p*-Value** |
| --- | --- | --- | --- | --- |
| **Cohort Characteristics** | | | | |
| N | 84 | 273 |  |  |
| Age, years | 68.92 ± 10.85 | 66.98 ± 9.04 |  | 0.102† |
| Male sex, n (%) | 34 (40.5) | 144 (52.7) |  | 0.065† |
| **SNpc Subregion Volume** | | | | |
| Limbic | 0.61 ± 0.11 | 0.46 ± 0.14 | 23.6 | **<0.001*** |
| Associative | 0.46 ± 0.12 | 0.30 ± 0.15 | 36.2 | **<0.001*** |
| Sensorimotor | 0.63 ± 0.12 | 0.42 ± 0.15 | 33.8 | **<0.001*** |

†*p*-value for age was calculated using an independent t-test, and that for sex was calculated using the χ² test.

**p*-value from age- and sex-adjusted analysis of covariance (ANCOVA).

Mean volumes are presented as raw values, and reduction (%) was calculated from the raw mean values.

SNpc, substantia nigra pars compacta; PD, Parkinson’s disease.

**Supplementary Table 7. Comparison of ^18^F-FP-CIT PET SBR between disease controls and patients with early-stage PD**

|  | **Disease Controls** | **Early-Stage PD** | **Reduction (%)** | ***p*-Value** |
| --- | --- | --- | --- | --- |
| **Cohort Characteristics** | | | | |
| N | 33 | 164 |  |  |
| Age, years | 64.94 ± 6.39 | 70.48 ± 6.33 |  | **<0.001†** |
| Male sex, n(%) | 11 (33.3) | 99 (60.4) |  | **0.008†** |
| **Mean SBR, cm^3^** | | | | |
| Dorsal striatum | 4.34 ± 0.60 | 2.04 ± 0.58 | 52.9 | **<0.001^*^** |
| Caudate nucleus | 4.00 ± 0.71 | 2.40 ± 0.79 | 40.0 | **<0.001^*^** |
| Caudate DA | 4.34 ± 0.73 | 2.54 ± 0.88 | 41.4 | **<0.001^*^** |
| Caudate VA | 4.19 ± 0.69 | 2.64 ± 0.80 | 37.1 | **<0.001^*^** |
| Caudate tail | 2.97 ± 0.70 | 1.71 ± 0.61 | 42.5 | **<0.001^*^** |
| Caudate body | 3.67 ± 0.76 | 2.19 ± 0.74 | 40.3 | **<0.001^*^** |
| Putamen | 4.50 ± 0.58 | 1.87 ± 0.57 | 58.4 | **<0.001^*^** |
| Putamen DA | 4.82 ± 0.63 | 2.10 ± 0.65 | 56.5 | **<0.001^*^** |
| Putamen VA | 5.21 ± 0.66 | 2.99 ± 0.81 | 42.7 | **<0.001^*^** |
| Putamen DP | 4.03 ± 0.57 | 1.32 ± 0.53 | 67.1 | **<0.001^*^** |
| Putamen VP | 4.09 ± 0.54 | 1.27 ± 0.53 | 68.9 | **<0.001^*^** |
| Pallidum | 1.00 ± 0.14 | 0.73 ± 0.13 | 26.8 | **<0.001^*^** |
| Anterior Pallidum | 1.09 ± 0.16 | 0.84 ± 0.16 | 23.4 | **<0.001^*^** |
| Posterior Pallidum | 0.88 ± 0.12 | 0.60 ± 0.11 | 31.8 | **<0.001^*^** |
| Ventral striatum | 4.66 ± 0.59 | 3.08 ± 0.80 | 33.9 | **<0.001^*^** |
| Substantia nigra | 0.91 ± 0.15 | 0.72 ± 0.14 | 21.2 | **<0.001^*^** |
| Subthalamic nucleus | 0.61 ± 0.13 | 0.55 ± 0.14 | 9.6 | **0.006^*^** |
| Dorsal raphe nucleus | 0.75 ± 0.22 | 0.71 ± 0.24 | 4.8 | 0.243* |
| Locus coeruleus | 0.42 ± 0.16 | 0.42 ± 0.15 | 1.3 | 0.503* |

†*p*-value for age was calculated using an independent t-test, and that for sex was calculated using the χ² test.

**p*-value from age- and sex-adjusted analysis of covariance (ANCOVA).

Mean volumes are presented as raw values, and reduction (%) is calculated from the raw mean values.

SBR, specific binding ratio; PD, Parkinson’s disease; DA, dorsal anterior; VA, ventral anterior; DP, dorsal posterior; VP, ventral posterior.
